# Temporal Dynamics of Serum Perforin and Granzyme during the Acute Phase of SARS-CoV-2 Infection

**DOI:** 10.3390/vaccines11081314

**Published:** 2023-08-01

**Authors:** MD Tazikur Rahman, Sukhyun Ryu, Chiara Achangwa, Joo-Hee Hwang, Jeong-Hwan Hwang, Chang-Seop Lee

**Affiliations:** 1 Department of Medical Science, Jeonbuk National University Medical School, Jeonju 54896, Republic of Korea; tazikurrahmanquanak65@gmail.com; 2 Department of Preventive Medicine, Konyang University College of Medicine, Daejeon 35365, Republic of Koreaciaraacha@gmail.com (C.A.); 3 Department of Internal Medicine, Jeonbuk National University Medical School, Jeonju 54896, Republic of Korea; zany78@naver.com (J.-H.H.); smilehwang77@hanmail.net (J.-H.H.); 4 Research Institute of Clinical Medicine of Jeonbuk National University—Biomedical Research Institute of Jeonbuk National University Hospital, Jeonju 54896, Republic of Korea

**Keywords:** perforin, granzyme, SARS-CoV-2, cytotoxic T cell

## Abstract

Background: As many SARS-CoV-2 infections are asymptomatic, it could be useful to be able to determine how much time has passed since infection. We explored the changes in the temporal levels of T cell-related proteins (including perforin and granzymes) in the sera of patients with SARS-CoV-2 infection using a commercially available assay. Methods: This study enrolled 36 patients infected with SARS-CoV-2 and 20 healthy control participants. Blood samples were collected at three different times based on the number of days since symptom onset (early phase: 1–5 days, mid-phase: 6–10 days, late phase: 11–18 days). We assessed the temporal changes in the serum levels of perforin and granzymes in patients with SARS-CoV-2 infection by comparing the results with those obtained in the healthy control group. Results: We identified a significantly low level of perforin in the early phase of SARS-CoV-2 infection (*p* < 0.01), which was restored to normal during the mid- and late phases of the infection. However, there was no difference in the temporal change in the level of granzymes in SARS-CoV-2-infected patients compared to the healthy control group. Conclusions: This finding suggests that SARS-CoV-2 infection paralyzed the perforin expression in the early period immediately after infection. Thus, serum perforin is a potential marker for identifying the acute phase of SARS-CoV-2 infection.

## 1. Introduction

Coronavirus disease 2019 (COVID-19) is an infectious disease caused by the severe acute respiratory syndrome coronavirus 2 (SARS-CoV-2). It is well demonstrated that many individuals with the SARS-CoV-2 infection remain asymptomatic [1,2]. Since active surveillance and control measures have been relaxed in many countries [3,4,5], people infected with SARS-CoV-2 who are asymptomatic or have only mild illness often do not get tested. Therefore, it is becoming more challenging to identify the clinical progress of COVID-19 during the period immediately following SARS-CoV-2 infection.

Measuring the cytotoxic T cell response after viral infection may help to assess the viral activation and predict clinical progress. Therefore, it can provide useful information about the timing of the infection. However, the levels of virus-specific T cells are patient-specific, which means that their measurement and analysis require abundant resources [6].

Perforin and granzyme, a pore-forming cytolytic molecule, are released by CD8^+^ T cells to disrupt the membranes of the target cells [7,8]. The levels of perforin and granzymes often increase in various infectious disease conditions, including viral and bacterial diseases [9,10]. These proteins can be obtained from a venous sample and are easily evaluated using a commercially available assay. Therefore, we assumed that the serum perforin and granzyme levels could be used as a proxy measure to assess the cytotoxic T cell response dynamics and estimate the time that elapsed since infection [6,11].

To identify the temporal marker of the period after infection, we investigated the serum levels of perforin and granzymes A and B at different times after SARS-CoV-2 infection among patients.

## 2. Materials and Methods

### 2.1. Patients and Data Collection

In total, 36 patients infected with SARS-CoV-2 were enrolled following admission to a tertiary hospital in South Korea between April 2020 and March 2021. To identify the SARS-CoV-2 infection, we obtained nasopharyngeal swab samples from the patients. Then, the infection was confirmed using a real-time reverse polymerase chain reaction. Since the infection process is often unobserved, the time after infection is often calculated using a clinical measure, such as symptom onset [12]. We collected blood samples at three different times based on the number of days that elapsed since symptom onset (early phase: 1–5 days, mid-phase: 6–10 days, late phase: 11–18 days). Furthermore, to identify the effect of the severity of the disease on the immune response, the severity was graded at the time of the first sample collection according to the World Health Organization 10-point clinical progression score, which is often used to assess the severity of patients with emerging infectious diseases, including COVID-19 [13]. Based on the clinical severity score, we identified 28 patients in the mild group (score 1–5) and eight patients were placed in the moderate–severe group (score of six to nine). To compare the level of perforin and granzyme A and B with the healthy control group, twenty healthy control participants (aged 24–58 years) were enlisted from the local population and were screened to confirm the absence of any infection (including SARS-CoV-2 using real-time reverse polymerase chain reaction) and/or other underlying diseases. All the study participants were unvaccinated against SARS-CoV-2 at the time of the study.

### 2.2. Patient Consent

All the study participants provided written informed consent, and all the study procedures were approved by the Institutional Review Board (IRB) of Jeonbuk National University Hospital (IRB registration number 2020-02-050).

### 2.3. Blood Serology

The early, mid- and late phase serum levels of perforin and granzymes A and B were assessed using a double antibody-based sandwich enzyme-linked immunosorbent assay (ELISA). Human perforin and granzymes A and B were analyzed in the ELISA Kit (Novus and Ray Biotech, Peachtree Corners, GA, USA) available from the Seoul Clinical Laboratories (SCL Healthcare, Seoul, Korea). A more detailed description of the assay has been described elsewhere [14,15,16].

### 2.4. Statistical Analyses

We compared the levels of perforin and granzymes A and B during different periods of infection and in different severities of disease with the healthy control group using a two-sided *t*-test or Mann–Whitney U-test where appropriate for the normal distribution or non-normal distribution of the data, respectively. In all the analyses, the perforin and granzyme A and B levels were log 10 transformed to reduce the variability of the data, including outlying observations, and a *p*-value < 0.05 was considered to indicate a statistical significance. All the statistical analyses and generating graphs were performed in R (version 4.0.1 for Windows, R Foundation for Statistical Computing).

## 3. Results

**Figure 1:**
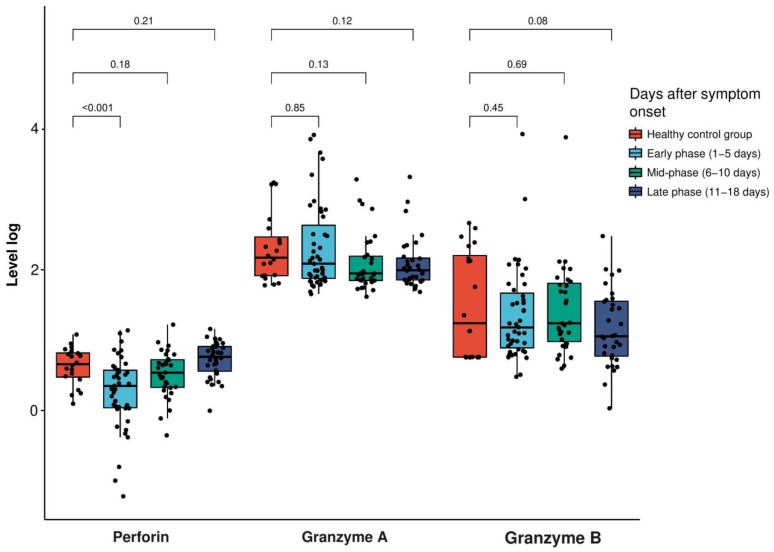
Extracellular levels of perforin in log(ng/mL) and granzyme A and B in log(pg/mL) in the patients with SARS-CoV-2 infection and the healthy control participants at the early, mid and late phases (early phase: 1–5 days, mid-phase: 6–10 days, late phase: 11–18 days after symptom onset). The box plot indicates the position of the interquartile ranges and median values. The significance was determined via a *t*-test or Mann–Whitney U-tests where appropriate, and a *p* < 0.05 was considered statistically significant.

The mean age of the 36 patients was 50 years, and 45% and 56% of the study population were males and females, respectively. The most common symptoms were fever (56%), myalgia (44%) and cough (33%) (Table 1). The level of perforin was significantly reduced (*p* < 0.001) during the early phase of SARS-CoV-2 infection (mean: 2.81 ng/mL, standard deviation (SD): 2.51 ng/mL) compared to the healthy control group (mean: 5.03 ng/mL, SD: 2.78 ng/mL). After the mid-phase of the infection, the perforin level was not significantly different from that of the healthy control group (*p* = 0.85 for mid-phase and *p* = 0.45 for late phase, respectively). However, the granzyme A and B levels during all the phases of infection showed no significant difference compared to the healthy control group (Figure 1 and Table 2).

**Figure 2:**
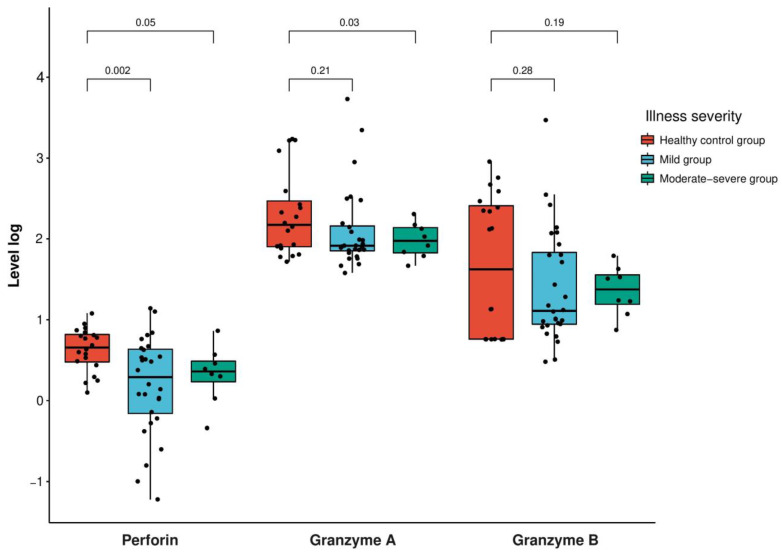
Expression levels of perforin in log(ng/mL) and granzyme A and B in log(pg/mL) in the patients with SARS-CoV-2 infection according to the severity (mild group, scoring 1–5; moderate–severe group, scoring 6–9 according to the World Health Organization’s 10-point clinical progression score) compared with the healthy control participants. The box plot indicates the position of the interquartile ranges and median values. The significance was determined via a *t*-test or Mann–Whitney U-tests where appropriate, and a *p* < 0.05 was considered statistically significant.

Furthermore, the level of perforin was significantly lower in the mild group compared to the healthy control group (*p*-value = 0.002), while the moderate–severe groups did not show a significant decrease (*p*-value = 0.05) (Figure 2, Table 2 and Supplementary Materials).

## 4. Discussion

**Figure 3:**
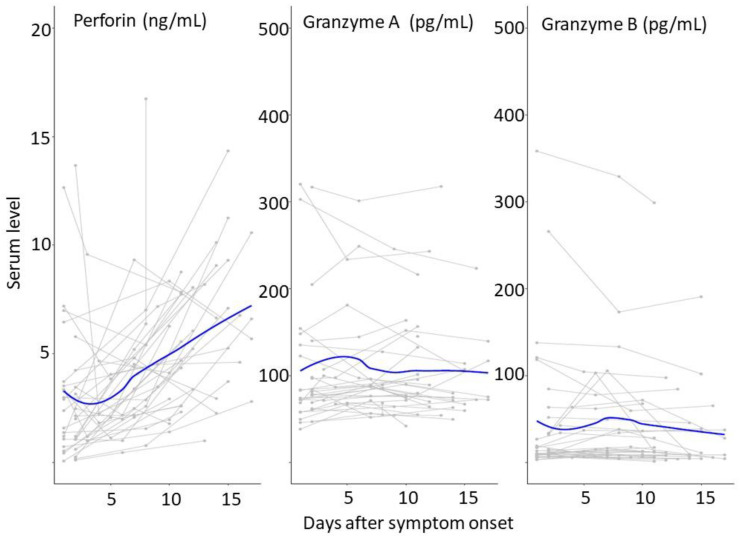
Temporal patterns of serum perforin and granzymes. Serum perforin and granzyme A and B were measured from the patients infected with SARS-CoV-2. The serums were obtained from the different days after their symptom onset. The thick blue lines indicate the trend of the serum perforin and granzyme A and B levels using smoothing splines.

Our findings showed a decreased level of perforin during the early phase after SARS-CoV-2 infection. The trend line for perforin from each individual also indicated a low level of perforin during the early period of infection (Figure 3). One of the possible explanations for a decreased level of serum perforin during the early infection phase was high perforin consumption at the intracellular level [17]. Another possible reason for decreased serum perforin was that the SARS-CoV-2 infection paralyzed the CD8^+^T cytotoxic functions and decreased the expression of perforin in the early phase of infection [18]. The low level of perforin was likely a result of defective cell-mediated immunity, which is a crucial defense against viral infection, and led to persistent SARS-CoV-2 (i.e., long COVID) [19]. We identified that the median level of perforin returned to normal after the mid-phase of the infection. This finding was similar to a previous Korean study that reported that increased perforin levels were observed during the third week of SARS-CoV-2 infection compared to the first week of the infection [8]. Another study also reported an increased level of serum perforin compared to the healthy control group during the second and third weeks of SARS-CoV-2 infection [17]. This finding was likely due to the disruption of the acid–base homeostasis in the acute phase of SARS-CoV-2 infection (i.e., acidosis in the patients with SARS-CoV-2 infection likely inhibited the release of perforin and blocked its activity) [20].

We identified that there was no significant difference in the levels of the granzymes during the different periods of infection compared to the healthy control groups. This finding was comparable to a previous study where granzymes increased during the third week (more than 21 days) of SARS-CoV-2 infection compared to the first week [8]. Therefore, the level of granzymes is unlikely to provide helpful information for the timing of SARS-CoV-2 infection during the acute phase after the infection.

A study demonstrated a tendency for decreased levels of intracellular perforin after the infection due to cytotoxic functional inactivity [18]. However, this study did not include information about the timing of infections and did not demonstrate whether the findings were statistically significant. Another study described statistically increased levels of intracellular perforin compared to the healthy control [21]. However, the timing of the extraction of the specimens was not demonstrated. Previous studies also demonstrated that the cell-mediated immune function, including the perforin expression, was statistically reduced for the elderly (above 70-year-old age) [22,23] and age-matched males. However, we did not include the elderly in the patients or the heathy control group. Furthermore, we did not identify a statistically significant difference in the number of males between the two groups. Therefore, the strength of our study was that it provided the temporal kinetics of perforin and granzymes A and B in the acute phase of SARS-CoV-2 infection.

There were some limitations to our study. First, this was a single-center study with a small sample size, which limited support for the generalization of our findings. Second, we did not perform flow cytometry to demonstrate whether the activated T cells were expressing perforin or granzyme A and B. However, perforin and granzymes are easy-to-measure serological markers of T cell viral activation [4]. Third, due to the limited specimens, we did not explore the correlation of perforin and granzymes with the SARS-CoV-2 RNA viral loads and immune cell numbers. An additional study that can disentangle and examine the correlation is warranted. Fourth, asymptomatic and severe patients with SARS-CoV-2 infection were not included in the study. Last, the time of symptom onset was used as a proxy for the time of infection. Therefore, the difference between the two may have affected our classification of the infection phases.

In conclusion, the serum level of perforin decreased during the early phase of SARS-CoV-2 infection. This finding indicates that the serum perforin level can provide useful clinical information as a temporal kinetic marker of the early period of SARS-CoV-2 infection. Further study that includes the cytokine associated with the T cell activation and the impact of COVID-19 vaccinations on the dynamics of perforin and granzymes using a large sample size is warranted.

## Figures and Tables

**Table 1:** The demographic and clinical characteristics of the patients with SARS-CoV-2 infection.

Characteristics	Overall (*n* = 36)	Mild Group (*n* = 28)	Moderate–Severe Group (*n* = 8)	*p*-Value
Age, y; mean ± SD	50.4 ± 17.5	46.2 ± 17.2	65.3 ± 8.7	0.005
Sex, *n* (%)				
Male	16 (44.44)	11 (39.29)	5 (62.50)	0.32
Female	20 (55.55)	16 (57.14)	3 (37.50)	
Comorbidities, *n* (%)				
Cardiovascular disease *	2 (5.60)	0	2 (25.00)	
Cerebrovascular disease ^¥^	1 (2.80)	1 (3.57)	0	
Liver disease ^€^	1 (2.80)	0	1 (12.50)	
Diabetes mellitus	4 (11.11)	3 (11.00)	1 (12.50)	
Hypertensive disorder	7 (19.40)	4 (14.30)	3 (37.50)	
Solid tumor ^₮^	4 (11.11)	3 (11.00)	1 (12.50)	
Clinical signs and symptoms, *n* (%)				
Fever	20 (55.60)	14 (50.00)	6 (75.00)	
General weakness	4 (11.11)	2 (7.14)	2 (25)	
Myalgia	16 (44.40)	10 (35.71)	6 (75.00)	
Cough	12 (33.30)	7 (25.00)	5 (62.50)	
Sputum	5 (13.90)	3 (10.71)	2 (25.00)	
Sore throat	3 (8.30)	3 (10.71)	0	
Dyspnea	1 (2.80)	0	1 (12.50)	
Anorexia	3 (8.30)	3 (10.71)	0	
Anosmia	5 (13.90)	5 (17.86)	0	
Ageusia	5 (13.90)	5 (17.86)	0	
Headache	6 (16.70)	6 (21.43)	0	
Nausea/vomiting	1 (2.80)	1 (3.57)	0	
Laboratory values, median (IQR)				
White blood cell count, ×1000/mm^3^	4.49 (3.52–6.36)	4.25 (3.27–6.36)	5.04 (4.58–6.48)	0.46
Lymphocyte count, ×1000/mL	0.92 (0.73–1.52)	0.93 (0.73–1.48)	1.08 (0.69–1.54)	0.92
Platelet count, ×1000/mm^3^	194.5 (156.0–248.8)	202(170–249)	140 (119–243)	0.05
Aspartate aminotransferase, IU/L	29.0 (22.00–40.75)	28 (20.0–39.8)	32 (39.3–58)	0.05
Alanine aminotransferase, IU/L	23.50 (16.00–36.75)	20 (15.3–34.8)	25 (24.3–39.5)	0.13
Lactate dehydrogenase, IU/L	409.00 (353.00–639.50)	371 (342–528)	654 (504–987)	0.003
Creatinine, mg/dL	0.70 (0.58–0.91)	0.69(0.59–0.81)	0.95(0.59–1.24)	0.16
C-reactive protein, mg/dL	5.03 (0.83–18.18)	2.82 (0.82–12.8)	24.8 (2.14–42.5)	0.07
Procalcitonin, ng/mL	0.07 (0.05–0.14)	0.06(0.04–0.07)	0.14 (0.09–0.24)	0.09
Treatment, *n* (%)				
Remdesivir	4 (11.11)	1 (3.57)	3 (10.71)	
Dexamethasone	4 (11.11)	1 (3.57)	3 (10.71)	
Lopinavir/Ritonavir	25 (69.4)	18 (64.29)	6 (21.43)	
Hydroxychloroquine	22 (61.1)	15 (53.57)	6 (21.43)	
Outcomes				
Hospital stay duration, median (IQR)	15.50 (12.00–22.25)	15.00 (12.00–21.25)	19.5 (14.25–25.25)	0.37
Intubation/Ventilation, *n* (%)	0 (0)	0 (0)	0 (0)	
Intensive care unit, *n* (%)	1 (2.80)	0 (0)	1 (12.50)	
Nasal high flow oxygen, *n* (%)	2 (5.60)	0 (0)	2 (25)	

* Includes myocardial infarction and angina. ^¥^ Includes subdural hematoma. ^€^ Includes hepatitis C virus carriers. ^₮^ Includes colorectal cancer, choriocarcinoma, malignant neoplasm of the common bile duct, breast carcinoma and pancreatic carcinoma.

**Table 2:** Temporal changes of the serum levels of perforin, granzyme A and granzyme B in the SARS-CoV-2 patients and healthy control participants and their differences by the severity of illness.

	Healthy Controls (*n* = 20)	Time from Symptom Onset						Illness Severity			
		Early Phase (*n* = 36)	*p*-Value	Mid-Phase (*n* = 36)	*p*-Value	Late Phase (*n* = 36)	*p*-Value	Mild Group (*n* = 28)	*p*-Value	Moderate–Severe Group (*n* = 8)	*p*-Value
Perforin (ng/mL)	5.03 ± 2.78	2.81 ± 2.51	<0.01	3.78 ± 2.28	0.85	6.49 ± 3.55	0.45	3.40 ± 3.54	<0.01	2.74 ± 2.06	0.05
Granzyme A (pg/mL)	143.42 ± 96.27	107.38 ± 71.53	0.18	106.04 ± 63.73	0.13	108.85 ± 63.47	0.69	107.70 ± 81.95	0.21	106.81 ± 53.10	0.03
Granzyme B (pg/mL)	212.98 ± 147.57	46.33 ± 75.73	0.21	50.03 ± 67.86	0.12	36.57 ± 61.71	0.08	32.99 ± 41.07	0.28	20.96 ± 13.40	0.19

Note: The values were presented as the mean ± standard deviation. The *p*-values were estimated using a *t*-test or Mann–Whitney U-tests comparing each phase of the SARS-CoV-2 infection and severity with the healthy controls after the levels were transformed log 10. Based on the days after symptom onset, the period of infection was classified as follows: early phase, 1–5 days; mid-phase, 6–10 days; late phase, 11–18 days. Based on the clinical severity score from the World Health Organization, the disease severity was classified into mild and moderate–severe groups.

## Data Availability

The data that support the findings of this study are available from the corresponding author upon reasonable request.

## Supplementary Materials

The following supporting information can be downloaded at: https://www.mdpi.com/article/10.3390/vaccines11081314/s1, Table S1: Temporal changes of serum levels of perforin, granzyme A, and granzyme B in the mild group of SARS-CoV-2 patients and healthy control participants; Table S2: Temporal changes of serum levels of perforin, granzyme A, and granzyme B in the moderate-severe group of SARS-CoV-2 patients, and healthy control participants.
