# Supplementary material for: Temporal Dynamics of Serum Perforin and Granzyme during the Acute Phase of SARS-CoV-2 Infection
Source: Vaccines (Basel). 2023 Aug 1;11(8):1314. doi: 10.3390/vaccines11081314 (PMC10459539; doi:10.3390/vaccines11081314)
Supplement: Supplementary file 1 [file vaccines-11-01314-s001.zip › vaccines-2491417-supplementary.pdf]

Supplementary Materials

Table S1. Temporal changes of serum levels of perforin, granzyme A, and granzyme B in the mild group of SARS-CoV-2 patients and healthy control participants.

	Healthy controls (n=20)	Mild group (n=77)	P-value	Time from symptom onset					
				Early phase (n=28)	P-value	Mid Phase (n=24)	P-value	Late phase (n=25)	P-value
Perforin (ng/mL)	5.03 ±2.78	3.39 ±3.54	< 0.01	2.93 ± 2.51	<0.001	3.17 ± 3.20	0.06	4.14 ± 3.76	0.26
Granzyme A (pg/mL)	143.42 ±96.27	107.69 ±81.95	0.25	105.48 ± 92.68	0.48	93.41 ± 90.47	0.30	124.20 ± 112.29	0.19
Granzyme B (pg/mL)	212.98 ±147.57	32.81 ±41.06	0.28	25.94 ± 20.14	0.55	39.79 ± 24.08	0.92	35.71 ± 21.38	0.09

Note: Values were presented in mean ± standard deviation. P-values were estimated by the t-test or Mann-Whitney U tests comparing each phase of SARS-CoV-2 infection and severity with the healthy controls after the levels were transformed log 10. Based on days after symptom onset, the period of infection was classified as follows; Early phase, 1 – 5 days; Mid-phase, 6 – 10 days; Late phase, 11 – 18 days. Based on the clinical severity score from World Health Organization, disease severity was classified into the mild groups.

Table S2. Temporal changes of serum levels of perforin, granzyme A, and granzyme B in the moderate-severe group of SARS-CoV-2 patients, and healthy control participants.

	Healthy controls (n=20)	Moderate-severe group (n=23)	P-value	Time from symptom onset					
				Early phase (n=8)	P-value	Mid Phase (n=7)	P-value	Late phase (n=8)	P-value
Perforin (ng/mL)	5.03 ±2.78	2.74 ±2.06	0.05	2.49 ± 1.99	0.052	2.76 ± 2.42	0.63	2.94 ± 5.29	0.37
Granzyme A (pg/mL)	143.42 ±96.27	106.81 ±53.10	0.03	111.81 ± 51.10	0.04	93.72 ± 49.11	0.02	114.92 ± 53.96	0.053
Granzyme B (pg/mL)	212.98 ±147.57	20.96 ±13.40	0.19	18.79 ± 16.80	0.23	17.28 ± 14.71	0.35	26.79 ± 15.78	0.22

Note: Values were presented in mean ± standard deviation. P-values were estimated by the t-test or Mann-Whitney U tests comparing each phase of SARS-CoV-2 infection and severity with the healthy controls after the levels were transformed log 10. Based on days after symptom onset, the period of infection was classified as follows; Early phase, 1 – 5 days; Mid-phase, 6 – 10 days; Late phase, 11 – 18 days. Based on the clinical severity score from World Health Organization, disease severity was classified into the moderate-severe groups.
